# *Drosophila* neural stem cell division and human tumorigenesis: fail to divide (asymmetrically) and tumors…will conquer? 

**DOI:** 10.3389/fcell.2026.1866713

**Published:** 2026-07-03

**Authors:** Ana Carmena

**Affiliations:** Instituto de Neurociencias, Consejo Superior de Investigaciones Científicas/Universidad Miguel Hernández de Elche, Alicante, Spain

**Keywords:** asymmetric cell division, cancer stem cells, *Drosophila*, neuroblasts, tumorigenesis

## Introduction

In a recent work, we have shown that restoring asymmetric cell division (ACD) in glioblastoma (GBM) stem cells could contribute to restraining tumor expansion (Franco et al., 2025). Building on these findings and accumulating evidence that ACD regulation shapes tumor growth dynamics, we propose that ACD restoration could be explored as a potential differentiation therapy. Under this framework, a tumor—while difficult to entirely eradicate—would cease spreading or spread at a much slower rate.

## *Drosophila* neuroblasts: a classical paradigm for analyzing asymmetric stem cell divisions

**FIGURE 1:**
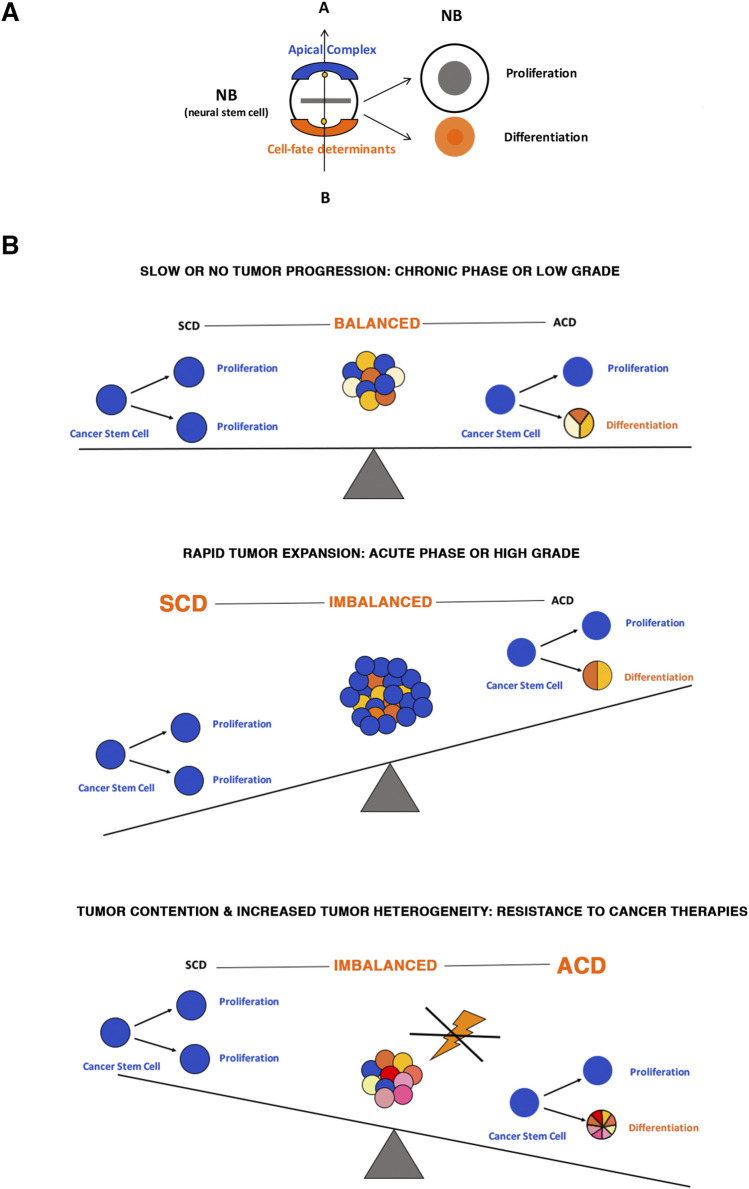
*Drosophila* neural stem cells (NBs) and cancer stem cells divide asymmetrically to promote differentiation. **(A)** Mitotic NBs are polarized (**(A)** apical; **(B)** basal); the mitotic spindle aligns along this axis of cell polarity and the cell-fate determinants (i.e., Numb and Brat), along with adaptor proteins, localize basally. Both events are tightly regulated by the apical complex, localized at the apical pole of metaphase NBs. In that way, only the most basal daughter cell receives the cell-fate determinants and it is committed to initiating a differentiation program. Yellow dots in the NB represent the centrosomes. **(B)** Cancer stem cells (CSCs) can divide symmetrically (SCD) to maintain the CSC pool and asymmetrically (ACD) to contribute to tumor heterogeneity. A balance between these two modes of cell division avoids tumor progression; however, an imbalance in these modes of cell division could contribute to a rapid tumor expansion (when ACD is compromised) or to a tumor contention but also increased tumor heterogeneity, with the consequent resistance to cancer therapies, (when ACD is favored).

The *Drosophila* neural stem cells of the central nervous system (CNS), called neuroblasts (NBs), represent an excellent model system for analyzing the process of ACD, which is a fundamental mechanism in development to generate cell diversity, and in the adult to regulate tissue homeostasis (Homem and Knoblich, 2012; Knoblich, 2008; Sunchu and Cabernard, 2020; Venkei and Yamashita, 2018). *Drosophila* NBs divide asymmetrically to give rise to two different daughter cells; one cell receives the so-called “cell-fate determinants” from the NB and will start a differentiation process, and another daughter does not inherit such determinants and keeps on proliferating, like the mother NB (Figure 1A). The asymmetric, basal localization of the cell-fate determinants–such as the cytoplasmic protein Numb and the translational repressor Brain tumor (Brat)– in the mother NB is key to the ACD process and must be tightly regulated by an intricate group of proteins known as the “apical complex”. This complex is asymmetrically located at the apical side of metaphase NBs and includes highly conserved proteins, such as the atypical protein kinase C (aPKC) and Par proteins (i.e., Par-6 and Par-3, called Bazooka -Baz-in *Drosophila*), among many others (Knoblich, 2008). Extrinsic signals secreted by a glial niche have also been shown to regulate the ACD of larval brain NBs, highlighting the relevance of the microenvironment in this process (de Torres-Jurado et al., 2022). ACD in mammals is quite conserved, despite the highest complexity in regulatory mechanisms, including also non-autonomous factors, such as niche signals, as well as interactions with stromal and immune cells (Daynac and Petritsch, 2017; Venkei and Yamashita, 2018; Knoblich, 2008; Inaba et al, 2012; Pham et al., 2015).

## Impaired asymmetric stem cell division as a driver of tumorigenesis

The first time that it was shown a connection between failures in the process of ACD and tumorigenesis was in *Drosophila* more than 20 years ago (Caussinus and Gonzalez, 2005). In this work, authors transplanted pieces of GFP-labeled larval brains mutant for different ACD regulators into the abdomen of wild-type flies observing, after weeks, the growth of big tumor masses. At around the same time, *Drosophila* genes originally identified as tumor suppressor genes were shown to be involved in ACD regulation, further strengthening the connection between ACD disruption and tumor growth (Albertson and Doe, 2003; Bello et al., 2006; Betschinger et al., 2006; Bowman et al., 2008; Gateff, 1978; Lee et al., 2006; Ohshiro et al., 2000; Peng et al., 2000). However, over these past years, we have learned that single mutations in most ACD regulators, with the exception of cell-fate determinants, do not cause tumor growth by their own; only when two or more ACD regulators are compromised tumorigenesis is induced (Carmena, 2018; Lee et al., 2006; Manzanero-Ortiz et al., 2021; Manzanero-Ortiz et al., 2024; Rives-Quinto et al., 2017). This illustrates how the relevance of ACD during development, tissue homeostasis and tumorigenesis has triggered the evolution of redundant mechanisms and synergistic effects, making ACD a highly regulated process. It is important to remark on the key influence of the tumor´s environment and the temporal window frame in which the tumor develops, when contemplating a compromised ACD-dependent induction of tumor-like overgrowth (Carmena, 2018).

As mentioned above, even though many molecular regulators of ACD are conserved in mammals, we have to be aware of the increased mechanistic and tissue-specific context complexity of ACD regulation in these systems. Despite this, over the past decades several studies have also strongly supported connections between impaired ACD and tumorigenesis in mammalian systems, including human tumors (Bajaj et al., 2015; Chen et al., 2014; Cicalese et al., 2009; Daynac et al., 2018; Franco et al., 2025; Hwang et al., 2014; Ito et al., 2010; Sugiarto et al., 2011; Tosoni et al., 2015).

## Cancer stem cells: “the roots that refuse to die”

The term of “cancer stem cell” (CSC) became very popular at the early 2000s, when it was proposed that at least some human tumors might be originated by these CSCs (Reya et al., 2001). The CSCs would represent a very small group of cells within the tumor but the only ones able to proliferate without control promoting tumor growth. Today we know that CSCs exist in many human tumors and that they have very particular additional properties, such as high capacity of DNA repair, high heterogeneity, and the ability to be in a quiescent stage, which make CSCs extremely resistant to chemotherapy and responsible for tumor relapse (Bajaj et al., 2020; Moore and Lyle, 2011; Reya et al., 2001; Rosen and Jordan, 2009; Yadav et al., 2020). Likewise, growing evidence regarding the controversial debate on the origin of CSCs points to cell sources other than normal stem or progenitor cells, such as fully differentiated cancer cells. These cancer cells, under the influence of multiple intrinsic and extrinsic signals, would be able to reprogram themselves to a stem cell state (Lee et al., 2025).

As all stem cells, CSCs can divide symmetrically, to maintain themselves by self-renewal, as well as asymmetrically, contributing in this way to tumor heterogeneity. An imbalance in the mode of division of CSCs could then promote rapid tumor expansion or increased tumor heterogeneity if symmetric or asymmetric divisions, respectively, are favored (Figure 1B) (Chao et al., 2024; Li et al., 2022).

Collectively, these CSC hallmarks underscore the importance of CSCs in driving tumor maintenance and expansion. Therefore, developing therapies that specifically target CSCs without altering normal stem cells remains a longstanding challenge. One promising approach to this is “differentiation therapy”.

## Differentiation therapy: a promising strategy for targeting cancer stem cell-driven tumor growth

Differentiation therapy aims to induce the differentiation of cancer cells, especially CSCs, instead of killing them, abrogating in that way their self-renewal properties and, consequently, reducing or even completely eliminating the tumor burden (de Thé, 2018; Zhu et al., 2023). The process and outcome of this therapy would represent a less aggressive treatment than chemo- or radiotherapy, as healthy non-cancer cells are much less affected. Differentiation therapy was very successfully implemented for the first time in acute promyelocytic leukemia (APL), which is nowadays almost completely cured by the combination of differentiation agents, particularly retinoic acid (RA) and arsenic (Lo-Coco et al., 2013). However, in the case of solid tumors the accomplishment of this therapy has been much more challenging than in leukemia, in part due to the increased genetic complexity of solid tumors. Despite this, some success has been achieved along the past years using differentiation therapy in these tumors (Bar-Hai and Ishay-Ronen, 2022; de Thé, 2018; Storm et al., 2016; Vogelstein et al., 2013).

## Restoring asymmetric cell division as differentiation therapy: a double-edged mechanism

In this context, we propose that promoting ACD in CSCs could serve as a differentiation therapy, as this type of division generates cells that are committed to differentiation. In our recent study, we found that restitution of human *RAP2A*, homolog of the *Drosophila* ACD regulator *Rap2l*, in glioblastoma (GBM) neurosphere cultures, decreases GBM stem cell (GSC) proliferation and stem cell markers, increasing ACD in these cells (Franco et al., 2025). Likewise, we observed that low levels of *RAP2A* in human GBM is associated with poor clinical prognosis. Thus, even though more investigation is necessary *in vivo* and in more GBM types, our data support that ACD restoration could contribute to contain tumor expansion. In fact, different studies over the past two decades have also shown promising results in this regard (Bajaj et al., 2015; Chen et al., 2014; Cicalese et al., 2009; Daynac et al., 2018; Franco et al., 2025; Hwang et al., 2014; Ito et al., 2010; Sugiarto et al., 2011; Tosoni et al., 2015). However, ACD has also been shown to be necessary to promote tumor heterogeneity, which makes the tumor more difficult to completely eliminate by radio- or chemotherapy. Therefore, different works have considered just the opposite approach to eradicate the tumor, suggesting that targeting ACD in CSCs would be the most favorable tactic for tumor elimination (Buss et al., 2024; Chao et al., 2023; Hitomi et al., 2021; Latifi et al., 2025; Samanta et al., 2023). It is worth to mention here that some reports have shown that tumor heterogeneity can be caused either by symmetric or asymmetric divisions and, in some cases, even with more contribution of symmetric divisions (Lathia et al., 2011; Ma et al., 2014).

Hence, enhancing ACD could be a two-sided coin: on one hand, it would reduce self-renewal CSC divisions restraining tumor progression and, on the other hand, it could contribute to increase tumor heterogeneity and resistance to cancer therapies. We therefore propose that the resolution to this conundrum may lie in achieving a “contained-tumor state” (i.e., a stable, non-progressive tumor condition), where tumor expansion is controlled without complete eradication (Figure 1B). Definitely, in order to consider ACD manipulation as an established strategy in tumor therapy, further research must be conducted and many more approaches implemented to tackle this challenging scenario.
